# Transitioning to Shorter, Oral Antimicrobial Therapy for Pelvic Osteomyelitis in Patients Living With Spinal Cord Injury

**DOI:** 10.1093/ofid/ofaf805

**Published:** 2026-01-06

**Authors:** Dhineli Perera, Ben Clegg, Ash Thomas, Sara Vogrin, Satwik Motaganahalli, Richard Clements, Caroline McFarlane, Estelle Petch, Andrew Nunn, Jason A Trubiano, Gemma K Reynolds

**Affiliations:** Department of Infectious Diseases, Austin Health, Heidelberg, Victoria, Australia; Department of Pharmacy, Austin Health, Heidelberg, Victoria, Australia; Victorian Spinal Cord Service, Austin Health, Heidelberg, Victoria, Australia; Department of Infectious Diseases, Austin Health, Heidelberg, Victoria, Australia; Department of Infectious Diseases, Doherty Institute, University of Melbourne, Melbourne, Victoria, Australia; Department of Infectious Diseases, Austin Health, Heidelberg, Victoria, Australia; Victorian Spinal Cord Service, Austin Health, Heidelberg, Victoria, Australia; Victorian Spinal Cord Service, Austin Health, Heidelberg, Victoria, Australia; Victorian Spinal Cord Service, Austin Health, Heidelberg, Victoria, Australia; Victorian Spinal Cord Service, Austin Health, Heidelberg, Victoria, Australia; Department of Infectious Diseases, Austin Health, Heidelberg, Victoria, Australia; Department of Infectious Diseases, Doherty Institute, University of Melbourne, Melbourne, Victoria, Australia; Department of Infectious Diseases, Austin Health, Heidelberg, Victoria, Australia; National Centre for Infections in Cancer, Peter MacCallum Cancer Centre, Melbourne, Victoria, Australia; Sir Peter MacCallum Department of Oncology, University of Melbourne, Melbourne, Victoria, Australia

**Keywords:** antimicrobial duration, antimicrobial stewardship, spinal cord injury, pelvic osteomyelitis, pressure injury

## Abstract

An 8-year prospective cohort study of pelvic osteomyelitis in patients living with spinal cord injury shows that 4- to 6-week, post-debridement, quinolone-sparing oral antimicrobial regimens were effective within multidisciplinary care. Clinical cure (89% at 12 months) remained high with shorter durations. These real-world findings support stewardship and inform prescribing and future trials.

Ninety-five percent of patients living with spinal cord injury (PLWSCI) will experience at least one pressure injury in their lifetime [1]. Between 17% and 58% of pelvic pressure injuries will progress to pelvic osteomyelitis (POM) [2]. All-cause mortality for POM in PLWSCI is 20%–25% [2].

The optimal duration of antimicrobial therapy for POM remains contentious [2–3]. A 2019 survey of infectious diseases (ID) physicians showed a lack of consensus approach and low confidence when treating POM [4]. To date, 7 retrospective cohort studies have evaluated treatment outcomes in spinal cord injury (SCI)–associated POM. While outcomes assessed were grossly heterogenous and lacked granular detail on antimicrobial selection and route, the majority supported 6–8 weeks of therapy [5–11]. At our center, SCI-associated POM is managed within a bespoke SCI antimicrobial stewardship program, which began in 2018 [12]. We aim to describe our prospective experience with POM in our quaternary SCI referral center.

## METHODS

### Design

A prospective cohort study of pressure injuries in PLWSCI, admitted between 2016 and 2023, was conducted at Austin Health (Melbourne, Australia). Austin Health provides statewide, quaternary referral spinal care services in a 26-bed acute SCI ward. Pressure injuries of any grade were included if they had surgical intervention and/or antimicrobial therapy. Pelvic pressure injuries were defined as those involving the ischial, sacral, or greater trochanteric regions. Diagnosis of POM was based on 1 or more of radiological (computed tomography/magnetic resonance imaging), histopathological, microbiological (≥1 positive intraoperative bone culture), or clinical criteria (eg, probe-to-bone). Outcomes were adjudicated by a multidisciplinary team comprising of a clinical nurse specialist, an SCI consultant, and two ID physicians. Patients under the age of 16, or those whose admission involved wound care alone, were excluded.

The primary outcome was the proportion of patients with POM who achieved clinical cure at 12 months post final surgery, acknowledging that debridement and subsequent flap were often performed as two-stage procedures in our center. Clinical cure was defined as the absence of unplanned surgical or antimicrobial intervention, or mortality attributed to POM-related infection within 3–12 months post final surgery. The first 3 months after definitive surgery incorporated an allied health–led graduated sitting program and comprehensive wound management to ensure optimal healing. Clinical cure assessment commenced at the completion of this program. Blind adjudication of this outcome was conducted by a third ID physician. Secondary outcomes examined the relationship between relapse, surgical approach, and antimicrobial prescribing patterns.

Inpatient management of pressure injuries for PLWSCI, including directing antibiotic therapy, was supported by a well-embedded multidisciplinary team [12]. The surgical approaches were grouped into 3 categories: debridement with direct closure, debridement and flap reconstruction, or healing with secondary intent. Two plastic surgeons performed all procedures during the study period. Surgical drains typically remained in situ for 3 weeks postoperatively; suction drain samples were not routinely analyzed.

Individual patient admissions were grouped in “episodes of definitive management” (EDM). A single EDM could span multiple individual hospital admissions pertaining to a single site of injury, if the patient had periods at home between debridement and definitive closure. Antimicrobial duration across the EDM was counted via reconciliation of electronic prescriptions and hospital medication administration and dispensing records, inclusive of those prescribed on discharge. Data on surgical interventions, microbiological findings, and outcomes were collected, with follow-up censured on 31 March 2025.

This study was approved by the Austin Human Research Ethics Committee (HREC/102374/Austin-2023).

### Statistical Analysis

Continuous variables are presented as median (interquartile range [IQR]), while categorical variables are presented as frequency (percentage). Groups (POM vs pelvic pressure injuries) were compared using the Mann–Whitney *U*-test and Fisher exact test. Trends over time were evaluated using Jonckheere–Terpstra test for trend. For the primary outcome of cure, logistic regression was performed. Standard errors were adjusted for clustering at the participant level to account for patients contributing to multiple EDMs during the study period. Due to low numbers of events, no multivariable analysis was performed. All analyses were conducted using Stata version 18.0 software (StataCorp, College Station, TX, USA).

## RESULTS

Of 205 EDMs (N = 147 patients) admitted over the study period for the management of pressure injuries, 124 (60%) had POM (Supplementary Figure 1). Characteristics of patients who developed POM in contrast to all those with stage I–III pressure injuries, excluding osteomyelitis, are detailed in Supplementary Table 1. The majority of patients with POM had thoracic injury levels (n = 85 [69%]). The median length of stay for an EDM for the POM cohort was 67.5 days (IQR, 34.5–120 days). The utilization rates of available diagnostic modalities for POM are described in Supplementary Table 2. Regarding surgical approach, debridement and flap reconstruction (45%) and debridement with direct closure (15%) was utilized for the majority of POM EDMs. The median antimicrobial duration per POM EDM was 46.5 days (IQR, 35–71 days), with a median intravenous duration of 14 days (IQR, 4–29.5 days) (Table 1). The median duration of intravenous antimicrobials significantly decreased over the study period, from a median of 16 days (IQR, 2–44 days) in 2016 to a median of 5 days in 2023 (IQR, 3–14 days; *P* = .025) (Supplementary Table 3), following introduction of our aforementioned antimicrobial stewardship rounds in 2018 [12].

**Table 1.** Spinal Cord Injury–Pelvic Osteomyelitis Patient Characteristics

Factor	Pelvic OM	Cured	Relapsed	OR (95% CI)	*P* Value
No. of patients	124	110	14	…	
Sex					
Male	107 (86)	95 (86.4)	12 (85.7)	0.95 (.23–3.98)	.941
Female	17 (14)	…	…	…	
Age, y, median (IQR)	54 (45–65)	54.5 (44–65)	54 (51–65)	0.99 (.96–1.02)	.554
Paralysis level					.75
Cervical	34 (27)	29 (26.4)	5 (35.7)	…	
Thoracic	85 (69)	76 (69.1)	9 (64.3)	…	
Lumbar	4 (3)	4 (3.6)	0 (0.0)	…	
Sacral	1 (1)	1 (0.9)	0 (0.0)	…	
CCI score, median (IQR)	1 (0.5–3)	1 (0–3)	1.5 (1–4)	1.00 (.77–1.30)	.981
Cumulative LOS, d, median (IQR)	67.5 (34.5–120)	67.5 (34–120)	67 (38–154)	1.00 (.99–1.01)	.895
Years since injury, median (IQR)	23 (11–35.5)	23 (10–35)	26 (13–40)	1.01 (.98–1.05)	.394
Diagnostic method for pelvic OM					
Histopathology: OM on bone (%)	22 (17.7)	…	…	…	
Microbiology: positive bone culture	81 (65.3)	…	…	…	
Radiologically proven OM	88 (71.0)	…	…	…	
MRI	62 (70.5)	…	…	…	
CT	19 (21.6)	…	…	…	
Other	7 (8.0)	…	…	…	
Primary site of pelvic pressure injury					
Greater trochanter	26 (21)	23 (21)	3 (21)	Ref	
Ischium	80 (64.5)	71 (64.5)	9 (64.3)	0.97 (.27–3.48)	.965
Sacrum	18 (14.5)	16 (14.5)	2 (14.3)	0.96 (.14–6.35)	.965
Wound chronicity prior to surgery					
≤6 mo	32 (25.8)	28 (25.5)	4 (28.6)	Ref	
>6 mo to <12 mo	37 (29.8)	33 (30.0)	4 (28.6)	0.85 (.18–3.90)	.833
≥12 mo	55 (44.4)	49 (44.5)	6 (42.9)	0.86 (.22–3.33)	.824
Prior flap at the same site					
Yes	29 (23.4)	23 (20.9)	6 (42.9)	2.84 (.93–8.63)	.066
Surgical approach					
Single or 2-stage flap	56 (45.2)	51 (46.4)	5 (35.7)	0.49 (.11–2.13)	.341
Debridement(s) and direct closure	18 (14.5)	15 (13.6)	3 (21.4)	Ref	
Healing with secondary intent	50 (40.3)	44 (40)	6 (42.9)	0.68 (.15–3.03)	.615
Organisms of interest					
*Staphylococcus aureus*	45 (36)	41 (37.3)	4 (28.6)	0.67 (.20–2.25)	.520
*Pseudomonas* spp	28 (22)	27 (24.5)	1 (7.1)	0.24 (.03–1.91)	.176
Total antimicrobial duration for EDM, d, median (IQR)	46.5 (35–71)	47 (35–73)	44.5 (41–49)	0.99 (.98–1.00)	.062
Single or 2-stage flap	47.5 (35–69.5)	…	…	…	
Debridement(s) and direct closure	48 (42–69)	…	…	…	
Healing with secondary intent	43.5 (32–78)	…	…	…	
Antimicrobial duration (categorized)					
≤6 wk	69 (56)	61 (56)	8 (57)	1.07 (.37–3.14)	.901
>6 wk	55 (44)	49 (44)	6 (43)	Ref	
Antimicrobial IV duration for EDM, d, median (IQR)	14 (4–29.5)	14 (4–27)	19 (7–43)	1.01 (.99–1.04)	.207

Data are presented as No. (%) unless otherwise indicated.

Patients who died within 365 days due to other reasons (not relapse) are included as being cured, as they had not had a relapse at their last visit (n = 8).

Abbreviations: CCI, Charlson Comorbidity Index; CI, confidence interval; CT, computed tomography; EDM, episodes of definitive management; IQR, interquartile range; IV, intravenous; LOS, length of stay; MRI, magnetic resonance imaging; OM, osteomyelitis; OR, odds ratio; PI, pressure injury; SCI, spinal cord injury.

Clinical cure was achieved in 89% of EDMs for POM at 12 months post final surgery (Table 1). Cure rates remained stable over the study period (*P* = .36). Table 1 summarizes baseline demographic factors, anatomical location, pressure injury chronicity, and microbiology in relation to clinical cure. Multivariable regression showed no association between relapse and age (*P* = .55), sex (*P* = .94), years since SCI (*P* = .39), or medical comorbidities (*P* = .98). Additionally, surgical approach showed no association with relapse rate (single or 2-stage flap: odds ratio [OR], 0.49 [95% confidence interval {CI}, .11–2.13]; *P* = .34). Total antimicrobial duration, as a continuous variable, was not associated with clinical cure (OR, 0.99 [95% CI, .98–1.00]; *P* = .062), nor was duration ≤6 weeks inferior to courses of >6 weeks’ duration (OR, 1.07 [95% CI, .37–3.14]; *P* = .901). All-cause mortality within the cured cohort was 7.3% at 1 year. There was one death in the relapse group related to SCI-associated POM.

**Figure 1.**
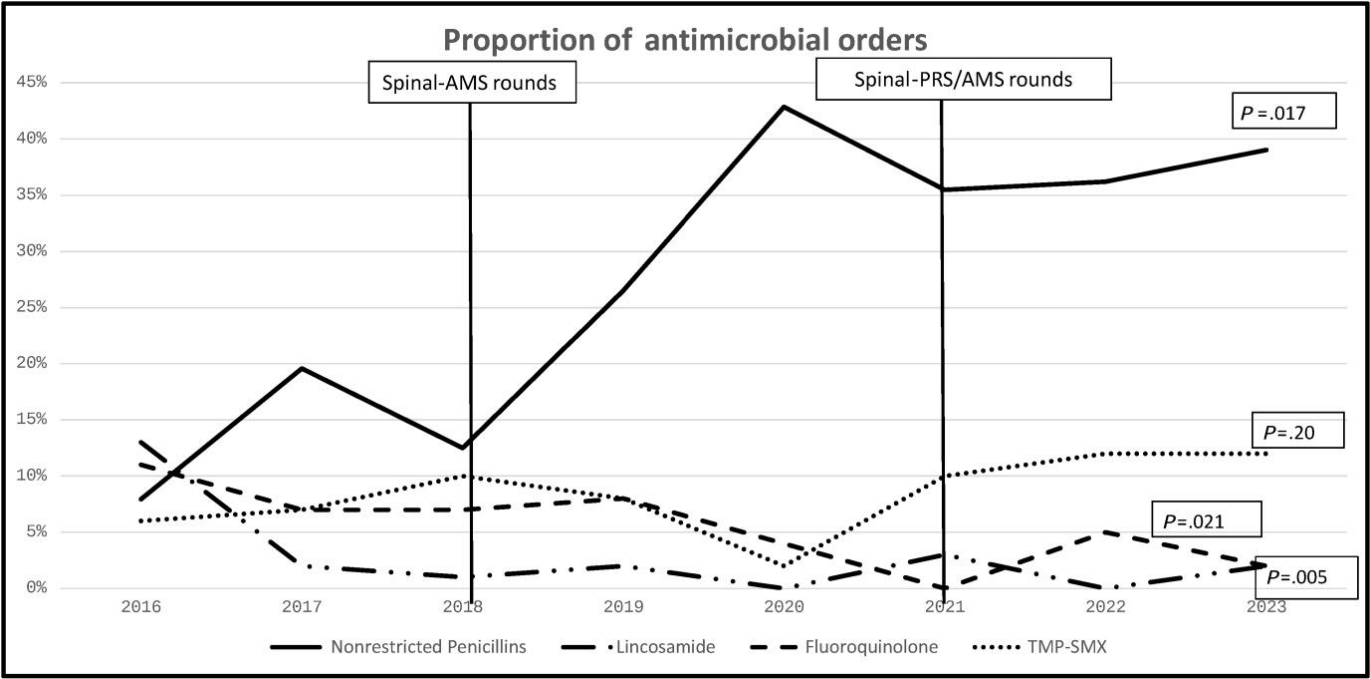
Selected antimicrobials of interest as a proportion of total antimicrobials orders over the study period. Proportion of antimicrobial orders by year for specific classes of antimicrobials as well as the statistical significance of their change over the study period. Further numerical detail can be found in Supplementary Table 2. Abbreviations: AMS, antimicrobial stewardship; PRS, plastic and reconstructive surgery; TMP-SMX, trimethoprim-sulfamethoxazole.

Over the study period, the three most frequently prescribed antimicrobials were amoxicillin-clavulanate (26%), cefazolin (13%) and trimethoprim-sulfamethoxazole (8%) (Supplementary Table 3). Use of narrow-spectrum penicillins increased significantly over the study period (*P* = .017; Figure 1). Prescribing of broad-spectrum antimicrobials such as carbapenems (2%) and fluoroquinolones (4%) was less frequent.

## DISCUSSION

We report a longitudinal, prospective cohort study of POM in PLWSCI. High clinical cure rates were achieved using standardized surgical approaches and multidisciplinary care. Our relapse rate was lower than shown in previous studies utilizing similar approaches, where relapse ranged between 17% and 35% [6, 9, 10]. Furthermore, cohort studies where surgical approach was either less standardized, or not clearly defined, showed higher relapse rates (55%–67%) [5, 7].

Our data expand on existing evidence by showing no increased relapse risk with antimicrobial durations ≤6 weeks in multivariate analysis, even after adjusting for surgical and demographic factors. This aligns with prior studies, which found no association between clinical cure and a longer duration of therapy [5–8, 11].

Most comparable to our cohort, Dinh et al. [9] and Marriott and Rubayi [10] explored utilization of a standardized surgical approach (debridement and flap reconstruction) and shorter durations of antibiotics (5–10 days and 4–6 weeks, respectively) in SCI cohorts at tertiary centers. Their subsequent relapse rates were higher than our cohort (31% and 17%, respectively), the contrast highlighted further when compared directly to our single or two-stage flap group, where a relapse rate of 9% was observed (Table 1). Our study extends their findings by demonstrating comparable outcomes despite lower use of quinolones and a higher utilization of narrow-spectrum β-lactams (Supplementary Table 3, Figure 1). The long-term impact of judicious antimicrobial prescribing for SCI patients frequently exposed to antimicrobial therapy as well as consistent diagnostic criteria for POM is not well characterized. Robust linkage of antimicrobial resistance patterns with stewardship practices is critical to inform safe, effective prescribing in this high-risk cohort.

In a recent state-of-the-art review, El Zein et al call for high-quality data pertaining to antimicrobial therapy choice and duration [2]. Our study provides granular insight into antimicrobial strategies for POM, highlighting successful use of primarily oral, short-course therapy. We account for the complexity and intermittency of treatment, capturing and quantifying antimicrobial use across multiple admissions as part of management for a single episode of POM. Study limitations include its single-center design and small sample size, which may limit statistical power to predict relapse. Length of stay for holistic management of these infections remains long, consistent with previous studies, despite shorter-course oral therapy [7, 10].

These findings suggest that shorter, predominantly oral antimicrobial therapy, combined with standardized surgical approaches and multidisciplinary care, may offer a viable alternative to prolonged intravenous therapy for POM. When considered alongside existing cohort studies, these results highlight genuine equipoise regarding POM diagnostic criteria and optimal antimicrobial selection and duration, supporting the need for a randomized trial in this complex population.

## Supplementary Material

ofaf805_Supplementary_Data

## Supplementary Data

Supplementary materials are available at *Open Forum Infectious Diseases* online. Consisting of data provided by the authors to benefit the reader, the posted materials are not copyedited and are the sole responsibility of the authors, so questions or comments should be addressed to the corresponding author.
