# Supplementary material for: Transitioning to Shorter, Oral Antimicrobial Therapy for Pelvic Osteomyelitis in Patients Living With Spinal Cord Injury
Source: Open Forum Infect Dis. 2026 Jan 6;13(1):ofaf805. doi: 10.1093/ofid/ofaf805 (PMC12798714; doi:10.1093/ofid/ofaf805)
Supplement: ofaf805_Supplementary_Data [file ofaf805_supplementary_data.docx]

**SUPPLEMENTARY FIGURES AND TABLES**

**Figure S1 – Spinal Cord Injury (SCI) – Pressure injury consort diagram**

31 admissions excluded due to not meeting inclusion criteria.

28 admissions merged with one or more admission to create an EDM

**Table S1 Patient and pressure injury characteristics**

| Factor | Pelvic OM (N=124) | Pelvic PI, no OM (N=61) | p-value |
| --- | --- | --- | --- |
| Sex (%)  Male  Female | 107 (86)  17 (14) | 55 (90)  6 (10) | 0.6 |
| Age, median (IQR) | 54 (45, 65) | 55 (43, 62) | 0.6 |
| Paralysis level  Cervical (%)  Thoracic (%)  Lumbar (%)  Sacral (%) | 34 (27)  85 (69)  4 (3)  1 (1) | 28 (46)  31 (51)  2 (3)  0 | **0.05** |
| CCI, median (IQR) | 1 (0.5, 3) | 1 (0, 3) | 0.58 |
| Cumulative LOS, median (IQR) | 67.5 (34.5, 120) | 43 (23, 87) | 0.011 |
| Years since injury, median (IQR) | 23 (11, 35.5) | 20 (8, 30) | 0.39 |
| Primary site of pelvic pressure injury  Greater trochanter (%)  Ischium (%)  Sacrum (%) | 26 (21)  80 (64.5)  18 (14.5) | 6 (9.8)  38 (62.3)  17 (27.9) | 0.038 |
| Wound chronicity prior to surgery  </= 6 months (%)  >6 months, < 12 months (%)  >/= 12 months (%) | 32 (25.8)  37 (29.8)  55 (44.4) | 27 (44)  8 (13)  26 (42.6) | **0.017** |
| Prior flap at the same site  Yes (%)  No (%) | 29 (23.4)  95 (76.4) | 8 (13.1)  53 (86.9) | 0.19 |
| Surgical approach (%)  Single OR Two-stage flap  Debridement/s and direct closure  Heal with secondary intent | 56 (45.2)  18 (14.5)  50 (40.3) | 26 (42.6)  11 (18.1)  24 (39.3) | 0.83 |
| Mortality  90-day (all cause)  1-year (all cause) | 4 (3.2)  9 (7.3) | 2 (3.3)  4 (6.6) | >0.99  >0.99 |
| Total antimicrobial duration for EDM, median (IQR) | 46.5 (35, 71) | 10 (5, 19) | <0.001 |
| Antimicrobial duration (categorized)  <6 weeks (%)  >6 weeks (%) | 69 (55.6)  55 (44.4) | 59 (96.7)  2 (3.3) | <0.001 |

SCI = Spinal cord injury, CCI = Charlson Comorbidity Index, OM = Osteomyelitis, PI = Pressure injury, LOS = Length of stay, EDM = Episodes of definitive management

P value from Mann-Whitney test or Fisher’s Exact test.

**Table S2 Availability of diagnostic modalities in patients with osteomyelitis**

| Combination of positive tests results | N (%) of patients |
| --- | --- |
| Histopathology + Radiology + Micro | 13 (10.4) |
| Histopathology + Radiology only | 4 (3.2) |
| Histopathology + Micro only | 8 (6.4) |
| Radiology + Micro only | 37 (29.8) |
| Histopathology only | 1 (0.1) |
| Radiology only | 34 (27.4) |
| Micro only | 23 (18.5) |
| Clinical only | 4 (3.2) |
| Total | 124 (100) |

**Table S3 Antimicrobial prescribing in POM (2016-2023)**

| Factor | 2016 | 2017 | 2018 | 2019 | 2020 | 2021 | 2022 | 2023 | p-value* |
| --- | --- | --- | --- | --- | --- | --- | --- | --- | --- |
| Episodes of definitive management (EDM) (N) | 17 | 10 | 18 | 21 | 13 | 9 | 18 | 18 |  |
| Total antimicrobial duration for EDM (days), median (IQR) | 60 (34, 71) | 32 (20, 94) | 48 (41, 92) | 48 (41, 72) | 46 (42, 56) | 42 (42, 53) | 42 (35, 57) | 47.5 (32, 78) | 0.44 |
| Antimicrobial duration (categorized)  <6 weeks (%)  >6 weeks (%) | 5 (29)  12 (71) | 6 (60)  4 (40) | 9 (50)  9 (50) | 11 (52)  10 (48) | 9 (69)  4 (31) | 6 (67)  3 (33) | 13 (72)  5 (28) | 10 (56)  8 (44) | 0.045 |
| Antimicrobial intravenous (IV) duration (days) for EDM, median (IQR) | 16 (2, 44) | 16 (4, 20) | 21 (14, 42) | 14 (8, 27) | 15 (9, 25) | 14 (8, 42) | 7.5 (3, 17) | 5 (3, 14) | 0.025 |
| Antimicrobial orders (N) (%) | 63 | 46 | 72 | 98 | 49 | 31 | 58 | 82 |  |
| Penicillins | 13 (21%) | 9 (20%) | 28 (39%) | 51 (52%) | 35 (71%) | 14 (45%) | 35 (60%) | 47 (57%) | <0.001 |
| Non-restricted | 5 (38%) | 9 (100%) | 9 (32%) | 26 (51%) | 21 (60%) | 11 (79%) | 21 (60%) | 32 (68%) | 0.017 |
| Restricted | 8 (62%) | 0 (0%) | 19 (68%) | 25 (49%) | 14 (40%) | 3 (21%) | 14 (40%) | 15 (32%) |  |
| Cephalosporins | 19 (30%) | 14 (30%) | 16 (22%) | 15 (15%) | 5 (10%) | 8 (26%) | 6 (10%) | 8 (10%) | <0.001 |
| Non-restricted | 14 (74%) | 11 (79%) | 12 (75%) | 8 (53%) | 2 (40%) | 6 (75%) | 2 (33%) | 4 (50%) | 0.041 |
| Restricted | 5 (26%) | 3 (21%) | 4 (25%) | 7 (47%) | 3 (60%) | 2 (25%) | 4 (67%) | 4 (50%) |  |
| Glycopeptide | 6 (10%) | 4 (9%) | 4 (6%) | 6 (6%) | 1 (2%) | 2 (6%) | 1 (2%) | 2 (2%) | 0.016 |
| Lincosamide | 8 (13%) | 1 (2%) | 1 (1%) | 2 (2%) | 0 (0%) | 1 (3%) | 0 (0%) | 2 (2%) | 0.005 |
| Metronidazole | 3 (5%) | 7 (15%) | 6 (8%) | 6 (6%) | 2 (4%) | 1 (3%) | 2 (3%) | 8 (10%) | 0.63 |
| Fluoroquinolone | 7 (11%) | 3 (7%) | 5 (7%) | 8 (8%) | 2 (4%) | 0 (0%) | 3 (5%) | 2 (2%) | 0.021 |
| Trimethoprim-Sulfamethoxazole | 4 (6%) | 3 (7%) | 7 (10%) | 8 (8%) | 1 (2%) | 3 (10%) | 7 (12%) | 10 (12%) | 0.20 |
| Tetracycline | 0 (0%) | 1 (2%) | 0 (0%) | 0 (0%) | 0 (0%) | 0 (0%) | 0 (0%) | 0 (0%) | 0.25 |
| Aminoglycoside | 2 (3%) | 0 (0%) | 1 (1%) | 0 (0%) | 1 (2%) | 0 (0%) | 0 (0%) | 1 (1%) | 0.38 |
| Carbapenems | 1 (1%) | 2 (4%) | 0 (0%) | 1 (1%) | 2 (4%) | 1 (3%) | 2 (3%) | 1 (1%) | 0.78 |
| Others | 0 (0%) | 2 (4%) | 4 (6%) | 1 (1%) | 0 (0%) | 1 (3%) | 2 (3%) | 1 (1%) | 0.69 |
| p-value* from Jonckheere-Terpstra test for trend  Restricted penicillins = intravenous (IV) amoxicillin-clavulanic acid, IV piperacillin-tazobactam  Non-restricted penicillins = oral amoxicillin-clavulanic acid, (IV/oral) flucloxacillin, oral amoxicillin, IV ampicillin, IV benzylpenicillin, phenoxymethylpenicillin oral  Restricted cefalosporins = IV ceftriaxone, IV ceftazidime, IV cefepime  Non-restricted cefalosporins = IV cefazolin, oral cefuroxime, oral cefalexin | | | | | | | | | |
